# Interleukin-15-Cultured Dendritic Cells Enhance Anti-Tumor Gamma Delta T Cell Functions through IL-15 Secretion

**DOI:** 10.3389/fimmu.2018.00658

**Published:** 2018-04-10

**Authors:** Heleen H. Van Acker, Sébastien Anguille, Hans De Reu, Zwi N. Berneman, Evelien L. Smits, Viggo F. Van Tendeloo

**Affiliations:** ^1^Laboratory of Experimental Hematology, Tumor Immunology Group (TIGR), Faculty of Medicine and Health Sciences, Vaccine & Infectious Disease Institute (VAXINFECTIO), University of Antwerp, Antwerp, Belgium; ^2^Division of Hematology, Antwerp University Hospital, Edegem, Belgium; ^3^Center for Cell Therapy and Regenerative Medicine, Antwerp University Hospital, Edegem, Belgium; ^4^Center for Oncological Research (CORE), Faculty of Medicine and Health Sciences, University of Antwerp, Antwerp, Belgium

**Keywords:** acute myeloid leukemia, γδ T cells, dendritic cell vaccination, immunotherapy, interleukin-15

## Abstract

Dendritic cell (DC) vaccination can be an effective post-remission therapy for acute myeloid leukemia (AML). Yet, current DC vaccines do not encompass the ideal stimulatory triggers for innate gamma delta (γδ) T cell anti-tumor activity. Promoting type 1 cytotoxic γδ T cells in patients with AML is, however, most interesting, considering these unconventional T cells are primed for rapid function and exert meaningful control over AML. In this work, we demonstrate that interleukin (IL)-15 DCs have the capacity to enhance the anti-tumoral functions of γδ T cells. IL-15 DCs of healthy donors and of AML patients in remission induce the upregulation of cytotoxicity-associated and co-stimulatory molecules on the γδ T cell surface, but not of co-inhibitory molecules, incite γδ T cell proliferation and stimulate their interferon-γ production in the presence of blood cancer cells and phosphoantigens. Moreover, the innate cytotoxic capacity of γδ T cells is significantly enhanced upon interaction with IL-15 DCs, both towards leukemic cell lines and allogeneic primary AML blasts. Finally, we address soluble IL-15 secreted by IL-15 DCs as the main mechanism behind the IL-15 DC-mediated γδ T cell activation. These results indicate that the application of IL-15-secreting DC subsets could render DC-based anti-cancer vaccines more effective through, among others, the involvement of γδ T cells in the anti-leukemic immune response.

## Introduction

Acute myeloid leukemia (AML) is a clonal neoplasm derived from myeloid progenitor cells with a high mortality rate (1, 2). Although most AML patients will achieve remission with induction chemotherapy, the majority of them will eventually relapse within 2 years. This is due to the persistence of residual leukemic (stem) cells, known as minimal residual disease (MRD) (3, 4). Therefore, eradication of MRD is a therapeutic priority in the treatment of AML. Despite recent advances in the biologic characterization of AML, standard treatment has remained largely unchanged. Hence, novel therapies to delay, or at best prevent, relaspe and prolong survival are urgently warranted. Given the high suitability of AML cells for immune intervention, dendritic cell (DC) vaccination is subject of intense examination in the MRD setting (5–7). DC vaccine strategies aim to generate an anti-tumor immune attack, by harnessing both the innate and adaptive arms of the immune system (6, 8). This dual action is of interest, considering that both arms of the immune system are unequivocally important and cooperate in the generation of an effective anti-tumor immune response (9, 10). For example, it has been shown that γδ T cells and CD8^+^ T cells synergistically target cancer stem cells (10).

γδ T cells, an unconventional T cell subset in the twilight zone between innate and adaptive immunity, have the ability to provide strong and sturdy anti-tumor responses (11, 12). Contrary to αβ T cells, activation of γδ T cells does not rely on antigen presentation by major histocompatibility complexes. On the other hand, γδ T cell activation can be induced by T cell receptor-dependent recognition of phosphoantigens, such as isopentenyl pyrophosphate (IPP). This intermediate of the mevalonate pathway is commonly overexpressed in AML blasts due to aberrations contributing to transformation (13, 14). Moreover, given their direct killing capability, antigen-presenting capacity and skill to mobilize other components of the immune system (8, 11), it is no coincidence that a recent meta-analysis of gene expression data from over 18,000 cancers, including hematological malignancies, identified the presence of γδ T cells to be the most significant factor associated with favorable prognosis (15). In line with this, γδ T cells exert meaningful control over AML (16). Harnessing γδ T cells by a DC vaccine is therefore an interesting approach to tackle MRD and to maximize the anti-tumor potency of vaccination [recently reviewed (8)]. However, little is known about DC-mediated γδ T cell activation and the widely used DC vaccines, that are commonly referred to as interleukin (IL)-4 DCs, have been found to be largely ineffective at stimulating γδ T cells (8).

IL-4 DCs are generated by stimulating monocytes with IL-4 and granulocyte-macrophage colony-stimulating factor for 5 days, followed by a maturation step of 1–2 days with a cytokine-based cocktail (17). We previously established a novel monocyte-derived DC generation protocol with improved potency by employing IL-15 and a toll-like receptor ligand, both generating strong immunostimulatory signals (17, 18). These so-called IL-15 DCs have already proven themselves superior to the classic IL-4 DCs based on their direct cytolytic activity against tumor cells and their capacity to stimulate adaptive (17) and innate (19) anti-tumor immunity. Moreover, CD8^+^ T cells and natural killer cells respond to the chemokine milieu created by IL-15 DCs, whereas IL-4 DCs are suboptimal in attracting effector lymphocytes (20). This is of importance considering that vaccine DCs can only perform their directing and activating functions *in vivo*, provided that the effector cells come in their vicinity. γδ T cells too are more prone to migrate towards IL-15 DCs (20), suggesting the possibility of interaction between both cell types.

In the current study, we characterized the IL-15 DC-mediated γδ T cell activation on both the phenotypic and functional level and investigated the potential mechanisms involved. With this, we focused on DC vaccination as a therapeutic approach to surmount MRD in AML patients.

## Materials and Methods

### Ethics Statement and Cell Material

This study was approved by the Ethics Committee of the Antwerp University Hospital (Edegem, Belgium) under the reference number B300201419756. Primary cells were isolated from buffy coats of healthy volunteers supplied by the Red Cross Blood Transfusion Center (Mechelen, Belgium). Immune cells of AML patients (Table 1) were obtained as residual material of the WIDEA DC vaccination trial (NCT01686334) or obtained *via* the division of Hematology of the Antwerp University Hospital. Informed consent was received from all patients for being included in the study. Peripheral blood mononuclear cells (PBMCs) were isolated by Ficoll density gradient centrifugation. γδ T cells were isolated using a negative (EasySep, Cologne, Germany) or positive (Miltenyi, Leiden, The Netherlands) immunomagnetic cell selection kit for cytokine production determination and cytotoxicity assays, respectively. γδ T cells isolated with the EasySep γδ T cell isolation kit were >90% pure, whereas with the anti-TCRγ/δ microbead kit of Miltenyi a purity of >95% was routinely obtained. The Burkitt’s lymphoma tumor cell line Daudi, a known target for γδ T cells, was kindly provided to us by the laboratory of Prof. Kris Thielemans (Free University of Brussels, Brussels, Belgium). The chronic myeloid leukemia cell line in blast crisis K562 was purchased from the American Type Culture Collection (Rockville, MD, USA) and the AML cell lines NB4 and THP-1 were obtained from the Deutsche Sammlung von Mikroorganismen und Zellkulturen (Braunschweig, Germany).

**Table 1:** Patient characteristics.

Patient number	UPN1	UPN2	UPN3	UPN4
Sex	F	F	M	F
Age at diagnosis	74	87	62	59
Who type	AML NOS, AML with maturation	AML with myelodysplasia-related changes	AML NOS, Acute monoblastic/monocytic leukemia	AML with t(8;21)(q22;q22);RUNX1-RUNX1T1
Prior treatment	Induction (eMICE); Consolidation (mini-ICE)	6 x Decitabine	Induction I (Ida-AraC); Induction II (Dauno-AraC)	Induction I (Ida-AraC); Induction II (Dauno-AraC)
Disease stage	CR1	CR1	CR1	CR1

*UPN1 and UPN2 were included in the WIDEA dendritic cell vaccination trial (NCT01686334). Inclusion criterion; completion of at least one cycle of induction chemotherapy and one cycle of consolidation chemotherapy, resulting in morphological complete remission. UPN3 and UPN4 were not included in the WIDEA dendritic cell vaccination trial (NCT01686334). Blood was drawn prior to the start of the first cycle of consolidation chemotherapy*.

*F, female; M, male; eMICE, elderly mitoxantrone + arabinoside-c + etoposide; mini-ICE, idarubicin + arabinoside-C + etoposide; Ida-AraC, idarubicin + arabinoside-C; Dauno-AraC, daunorubicine + arabinoside-c; CR1, first complete remission; R1, first relapse; PB, peripheral blood; UPN, unique patient number*.

### Dendritic Cell Culture

Monocyte-derived IL-15 DCs were prepared conforming to our previously described 48-hour culture protocol (18, 20). Positively selected CD14^+^ monocytes (Miltenyi) are cultured in Roswell Park Memorial Institute medium (Life Technologies, Merelbeke, Belgium) with 2.5% heat-inactivated human AB serum (Life Technologies) at a final concentration of 1–1.2 × 10^6^ cells/mL. To generate mature IL-15 DCs, a 28-hour differentiation step using GM-CSF (800 IU/mL; Life Technologies) and IL-15 (200 ng/mL; Immunotools, Friesoythe, Germany) is followed by maturation induction with R848 (3 µg/mL; Alexis Biochemicals, San Diego, USA), interferon (IFN)-γ (250 ng/mL; Immunotools), tumor necrosis factor (TNF)-α (2.5 ng/mL; Life Technologies), and prostaglandin E2 (1 µg/mL; Pfizer, Puurs, Belgium) for 20 hours. To collect 48-hour wash-out supernatant, IL-15 DCs are harvested, thoroughly washed, and reseeded in fresh medium at a concentration of 1 × 10^6^ cells/mL in low-absorbing polypropylene tubes. After 48 hours, cell-free supernatant is collected and frozen at −20°C, until further use.

### Co-Culture Setup

PBMCs, unless stated otherwise, were cultured with IPP (122 µM; Tebu-bio, Le-Perray-en-Yvelines, France), autologous IL-15 DCs, and/or tumor cells (K562, Daudi) at an effector-to-target cell (E:T) ratio of 1:(1:)10. Unstimulated cells were used as negative control. With regard to cultures with immune cells of AML patients (Table 1), the same culture setup was used let alone the use of peripheral blood lymphocytes (PBLs) instead of PBMCs.

### Immunophenotyping

γδ T cells were phenotyped after 48-hour co-culture using the following monoclonal antibodies (mAbs): γδ TCR-FITC (Miltenyi), CD86-FITC, CD80-PE, PD-1-PE, NKG2D-PE, CD16-PB, BTLA-BV421, γδ TCR-APC (Miltenyi), NKp30-AF647, and CD3-APC-H7. Unless specified otherwise, all mAbs were purchased from BD (Erembodegem, Belgium). Live/Dead^®^ Fixable Aqua Stain (Invitrogen, Merelbeke, Belgium) was used to exclude dead cells from analysis. Corresponding species- and isotype-matched antibodies were used as controls. The corresponding gating strategy can be retrieved from Figure S1 in Supplementary Material.

### Proliferation Assay

Proliferation of γδ T cells was quantified by the degree of 5,6-carboxyfluorescein diacetate succinimidyl ester (CFSE; Invitrogen) dye dilution. At different time points, γδ T cell proliferation was measured as the proportion of CFSE-diluted cells within the viable CD3^+^γδ^^TCR^+^ gate (Live/Dead^®^ Fixable Aqua Stain, CD3-PerCP [BD], γδ TCR-APC). Unstimulated CFSE-labeled cells served as a non-dividing control.

### Cytokine Production

IFN-γ, TNF-α, IL-4, IL-10, and IL-17 (BD) production by γδ T cells was assessed intracellularly after overnight co-culture. Cells were fixed and permeabilized using the Foxp3/Transcription Factor Staining Buffer Set (eBioscience, Vienna, Austria). The IL-15 concentration in 48-hour wash-out supernatant of IL-15 DCs was quantified using electrochemiluminescent detection on a SECTOR3000 [Meso Scale Discovery (MSD), Rockville, MD, USA].

### Defining Contact- and IL-15 Dependency

To examine the role of cell–cell contact, IL-15 DCs were separated from γδ T cells by a 0.4-µm pore size Transwell insert (Elscolab, Kruibeke, Belgium). In specific experiments, 10 µg/mL anti-IL-15 neutralizing mAbs (R&D, Minneapolis, Canada) or corresponding isotype control mAbs were added to investigate the involvement of IL-15. IL-15 DCs were incubated for 1 hour with the above mAbs before starting co-culture to ensure adequate blocking of IL-15. The mAbs remained present during the further course of the co-culture.

### γδ T Cell-Mediated Cytotoxicity Assays

In order to measure the tumoricidal capacity of (un)stimulated γδ T cells, a flow cytometric assay was setup as previously described with minor modifications (21–24). 0.2 × 10^6^ isolated γδ T cells were cultured for 24–36 hours in the presence or absence of IPP (122 µM) and/or autologous IL-15 DCs at a DC: γδ T cell ratio of 1:10 in 200 µL RPMI (Invitrogen) supplemented with 10% fetal bovine serum (FBS; Invitrogen). Subsequently, tumor cells (Daudi, K562, NB4, and THP-1) and fresh allogeneic primary AML blasts (UPNa/b) were labeled with CellTracker™ Blue CMF2HC Dye (Invitrogen) and served as “target cells”. They were added to the γδ cell ± DC (co-)cultures for 4 hours at an E:T ratio of 2:1 in a final volume of 300 µL RPMI + 10% FBS. Cultures with allogeneic AML material were stained with CD33/CD34 to discriminate between AML blasts and the rest of the PBMC fraction of UPNa/b. Cell death was quantified after Annexin-V-APC (BD) and propidium iodide (PI) staining. Specific target cell killing was calculated according to the formula: % killing = 100 – [(% Annexin-V^−^/PI^−^ target cells with γδ T cells/% Annexin-V^−^/PI^−^ target cells without γδ T cells) × 100].

### Statistics

All flow cytometry data were acquired on a FACSAria II flow cytometer (BD) and analyzed using FlowJo (v10; Treestar, Ashland, OR, USA). For statistics and illustrations, GraphPad Prism software (v5.0; San Diego, CA, USA) was used. To validate the Gaussian distribution of data sets, they had to pass Shapiro–Wilk test for normality. Dissimilarities were predefined to be statistically significant when *p* < 0.05. All data are depicted as means ± SE of the mean.

## Results

### IL-15 DCs Induce the Upregulation of Cytotoxicity-Associated and Co-Stimulatory Molecules on the γδ T Cell Surface, But Not Co-Inhibitory Molecules

First, phenotypic characterization of γδ T cells was performed after a 48-hour culture period, either unstimulated or with the addition of IPP and/or IL-15 DCs (Table 2; % and ΔMFI, Figure S2 in Supplementary Material; histogram overlays). γδ T cells exposed to IL-15 DCs were found to express higher levels of markers related with γδ T cell cytotoxicity (CD16) and co-stimulatory molecules (CD80, CD86). Further upregulation of CD16, NKp30, CD80, and CD86 was observed with the combination of IL-15 DCs with IPP. In contrast, the expression of the co-inhibitory receptors and exhaustion-associated molecules BTLA and PD-1 on γδ T cells were not or only limited influenced by IL–15 DCs. The addition of IPP, however, resulted both in a significant induction and upregulation of PD-1 on γδ T cells.

**Table 2:** γδ T cell phenotype of healthy donors after 48 hours of culture with IPP, IL-15 dendritic cells (DCs) or both.

%	Resting	IPP	IL-15 DCs	IPP + IL-15 DCs
CD16	12.36 ± 3.45%	15.39 ± 3.72%	16.99 ± 4.16%	17.26 ± 4.19%**
NKG2D	62.52 ± 11.40%	66.30 ± 10.94%	63.42 ± 11.20%	66.27 ± 11.18%
NKp30	9.328 ± 2.403%	15.95 ± 2.59%	17.16 ± 3.08%	20.70 ± 2.58%*
BTLA	47.78 ± 7.24%	46.30 ± 7.07%	46.48 ± 7.23%	42.35 ± 7.91%
PD-1	6.738 ± 1.545%	19.27 ± 2.37%**	11.42 ± 1.48%	20.84 ± 3.19%*
CD80	0.7833 ± 0.2737%	2.892 ± 1.174%	6.067 ± 2.033%*	9.115 ± 3.912%**
CD86	3.132 ± 0.937%	15.69 ± 6.63%	13.28 ± 2.71%	20.32 ± 4.49%***

**ΔMFI**	**Resting**	**IPP**	**IL-15 DCs**	**IPP + IL-15 DCs**

CD16	237.5 ± 103.3	296.1 ± 118.0	376.4 ± 162.0*	379.8 ± 174.2*
NKG2D	8750 ± 4599	8871 ± 4482	8786 ± 3931	9662 ± 4407
NKp30	58.38 ± 12.64	88.17 ± 10.82	98.00 ± 16.82	114.7 ± 12.5*
BTLA	80.33 ± 9.14	95.52 ± 41.69	83.70 ± 9.84	85.40 ± 18.97
PD-1	65.38 ± 9.20	174.2 ± 28.6*	108.0 ± 10.0	187.9 ± 32.8*
CD80	5.450 ± 1.743	35.37 ± 13.36	69.12 ± 20.72*	89.48 ± 36.14*
CD86	19.48 ± 4.62	59.77 ± 16.87	69.77 ± 9.63*	83.77 ± 8.15**

*ΔMFI, difference in mean fluorescence intensity between the marker and corresponding isotype control*.

Asterisks indicate a statistically significant difference in cell surface marker expression between stimulated γδ T cells and resting γδ T cells (*n* = 6; Friedman test with Dunn’s Multiple Comparison Test; *** *p* < 0.001, ** *p* < 0.01, and * *p* < 0.05).

### Soluble Factors Secreted by IL-15 DCs Promote γδ T Cell Proliferation

**Figure 1:**
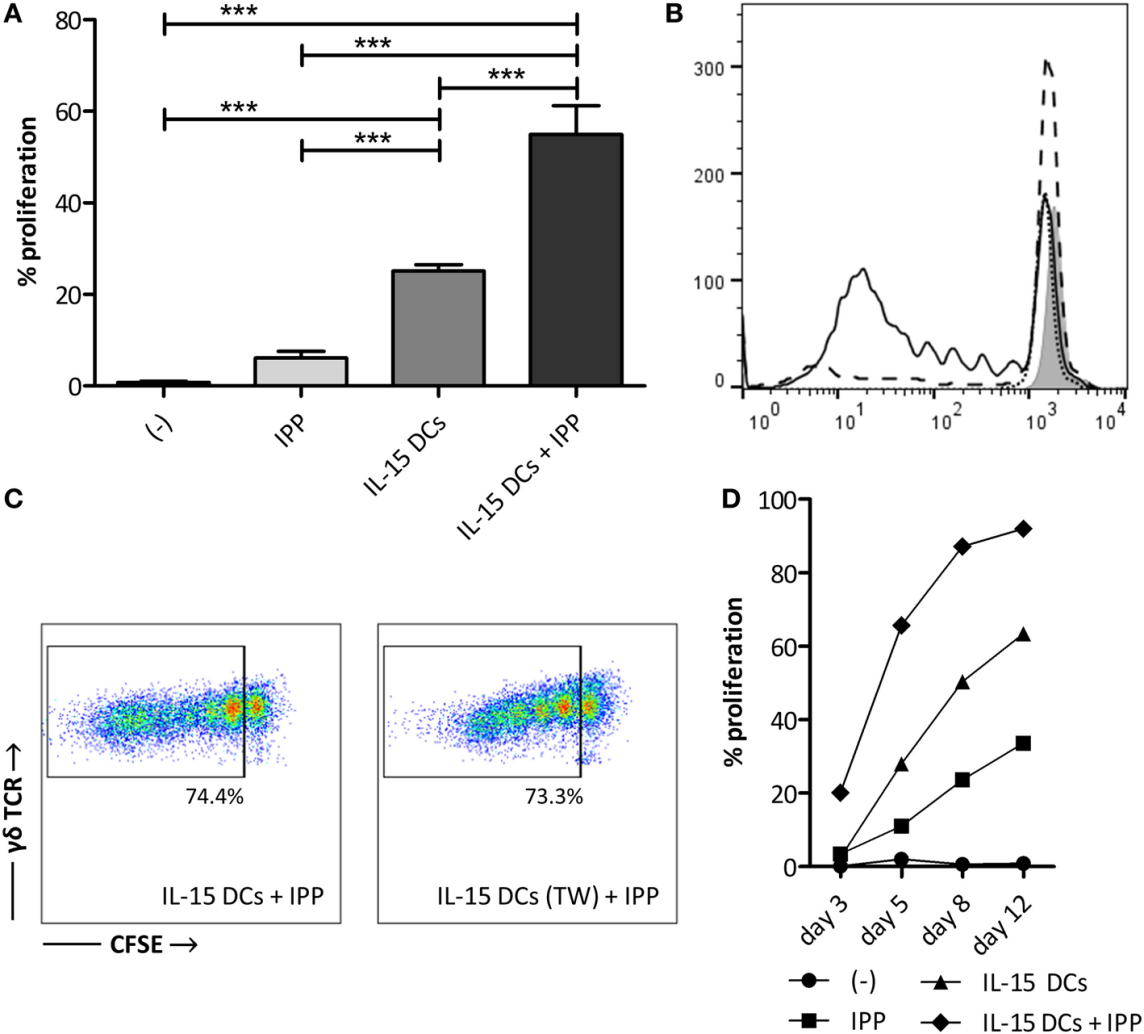
IL-15 dendritic cells (DCs) induce γδ T cell proliferation in a contact-independent manner. **(A)** γδ T cells in full peripheral blood mononuclear cell (PBMC) fraction were stimulated with isopentenyl pyrophosphate (IPP), IL-15 DCs (E:T ratio = 1:10) or IL-15 DCs + IPP for 5 days. Unstimulated PBMCs (−) were used as negative control. γδ T cell proliferation, as assessed by reduction in CFSE staining, was determined by flow cytometry. The percentage of cells to have undergone at least one cell division is shown (*n* = 12). One-way ANOVA with Bonferroni’s Multiple Comparison Test. **(B)** The histogram overlay shows CFSE dilution of viable γδ T cells within unstimulated PBMCs (filled gray), PBMCs stimulated with IPP (dotted line), IL-15 DCs (dashed line), and IL–15 DCs + IPP (full line) for one representative donor. **(C)** Dot plots of one representative donor (*n* = 3) showing the CFSE-dilution of proliferated γδ T cells in PBMCs stimulated with IL-15 DCs + IPP with and without IL-15 DCs in transwell (TW). **(D)** γδ T cell proliferation over time (experimental setup in line with panel A; *n* = 3). *** *p* < 0.001.

Next, we established the influence of IL-15 DCs on resting γδ T cells in full PBMC fraction through a standard 5-day proliferation assay (Figures 1A,B). IL-15 DCs induced significant γδ T cell proliferation, i.e. 25.13 ± 1.33% after 5 days. This proliferative response was doubled (54.98 ± 6.25%) in the presence of IPP. However, there was no clear proliferative reaction of the γδ T cells to IPP stimulation only after 5 days. When looking at a longer period, i.e. 8 and 12 days, IPP addition does result in γδ T cell proliferation (day 12; 33.60 ± 1.25%). Moreover, regarding these later time points, it is evident that the stimulatory effect of IL-15 DCs is relevant for the generation of high numbers of γδ T cells long term, namely 63.37 ± 8.81% γδ T cell proliferation after a 12-day co-culture (Figure 1D). To end, a similar degree of γδ T cell proliferation was observed after 5 days, irrespective of whether IL–15 DCs were separated from the PBMCs by a transwell membrane or not. This indicating that soluble factors secreted by IL–15 DCs were responsible for the observed proliferative response (Figure 1C).

### IL-15 DCs Stimulate IFN-γ Production in γδ T Cells in the Context of a Leukemic Environment

**Figure 2:**
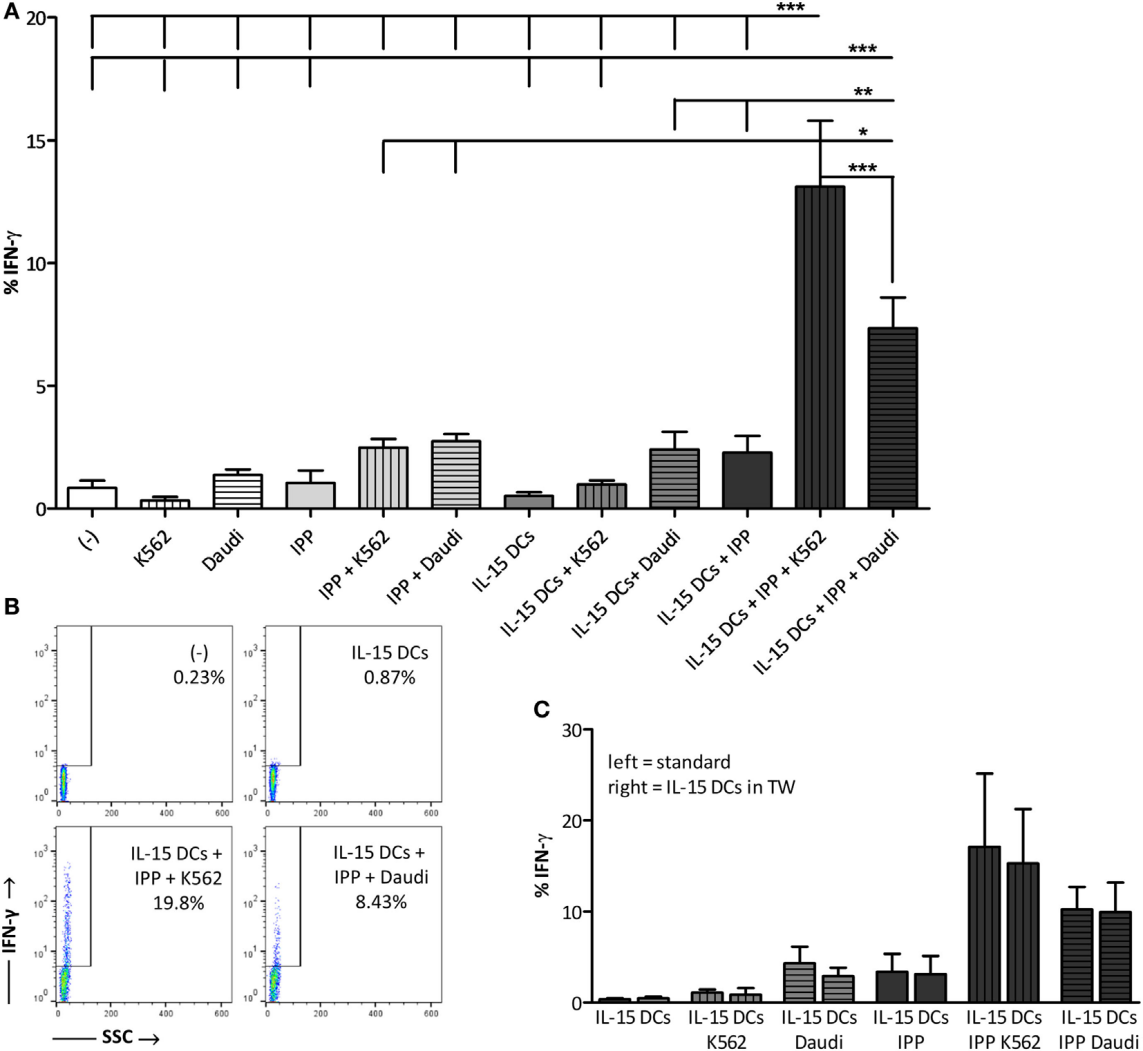
IL-15 dendritic cells (DCs) stimulate γδ T cell IFN-γ production in the presence of a leukemic environment through a contact-independent mechanism **(A)** γδ T cells in full peripheral blood mononuclear cell fraction were examined for IFN-γ production by intracellular staining after overnight stimulation with isopentenyl pyrophosphate (IPP), IL-15 DCs (E:T ratio = 1:10), tumor cells (E:T ratio = 1:10), and all possible combinations. Resting γδ T cells (−) were used as negative point of reference. Data are depicted as the mean of ten independent donors. One-way ANOVA with Bonferroni’s Multiple Comparison Test. **(B)** Representative FACS dot plots of one healthy donor out of 10 showing IFN-γ production by γδ T cells. **(C)** In parallel, the same experiment was carried out except that IL-15 DCs were separated from the cultures by 0.4-µm transwell (TW) inserts (*n* = 3). *** *p* < 0.001, ** *p* < 0.01, and * *p* < 0.5.

One of the desirable effector functions of γδ T cells in cancer immunotherapy is the secretion of pro-inflammatory cytokines, such as IFN-γ and TNF-α. Therefore, we measured the intracellular IFN-γ production in γδ T cells after overnight stimulation (Figures 2A,B). Stimulation of γδ T cells with IPP and/or tumor cells without DCs failed to trigger IFN-γ production in γδ T cells. IL-15 DCs alone also did not provide the necessary signals for IFN-γ production. On the other hand, when IL-15 DCs were added together with signals of malignancy (presence of IPP and tumor cells) a strong IFN-γ response was observed in γδ T cells. As shown in Figure 2A, stimulation with IL-15 DCs + IPP + tumor cell lines resulted in 13.12 ± 2.68% (for K562 cell line) and 7.35 ± 1.24% (for Daudi cell line) IFN-γ^+^ γδ T cells. TNF-α production was absent in all situations, as well as the T helper type 2 (Th2)-promoting cytokine IL-4, the Th17-promoting cytokine IL-17 (Th17), and the anti-inflammatory cytokine IL-10 (data not shown). In analogy with the induction of γδ T cell proliferation, the stimulation of IFN-γ production in γδ T cells by IL-15 DCs was found to be contact-independent (Figure 2C).

### Activation of γδ T Cells by IL-15 DCs Is Dependent on Soluble IL-15

**Figure 3:**
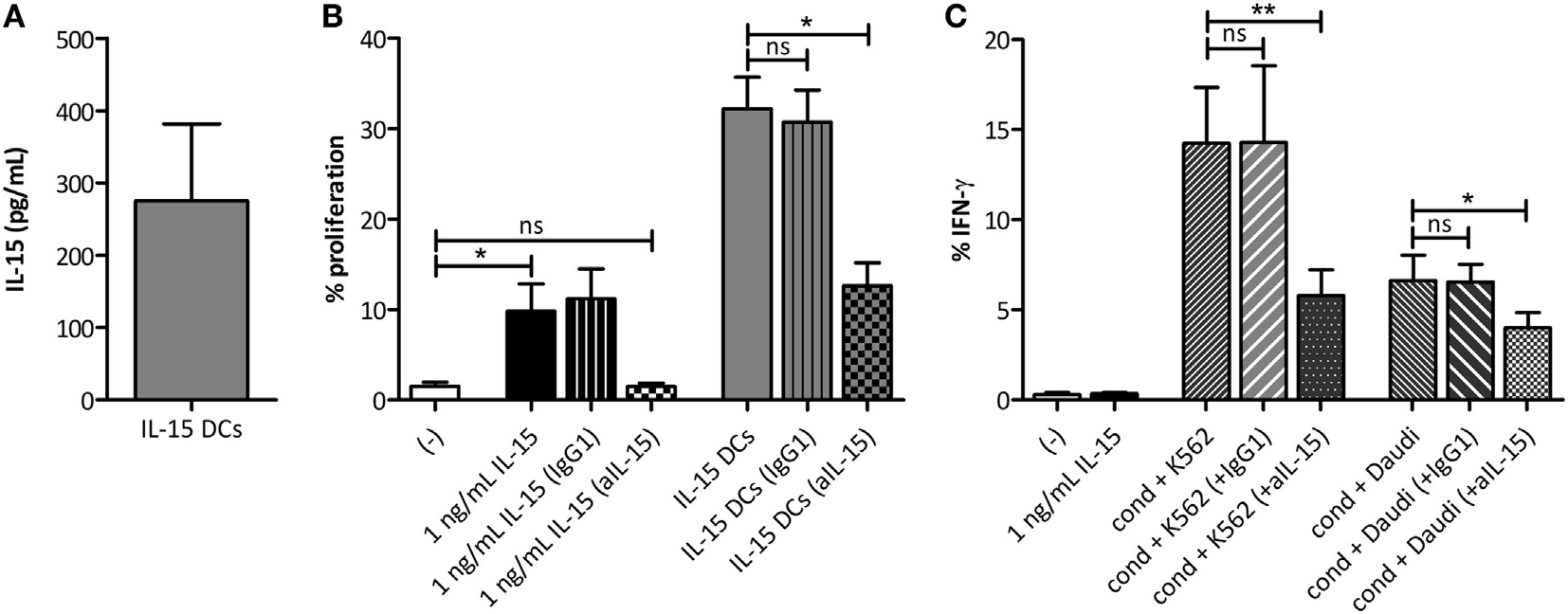
IL-15, secreted by IL-15 dendritic cells (DCs), provides an important signal for DC-mediated γδ T cell proliferation and IFN-γ production. **(A)** Representation of the IL-15 secretion level (pg/mL), as determined by Meso Scale Discovery immunoassay, in 48-hour wash-out supernatant of IL-15 DC cultures (1 × 10^6^ cells/mL; *n* = 3). **(B)** CFSE-labeled peripheral blood mononuclear cells (PBMCs) were co-cultured with 1 ng/mL recombinant IL-15 or IL-15 DCs (E:T ratio = 1:10). In certain conditions, neutralizing IL-15 monoclonal antibodies (mAbs) (aIL-15) or isotype control mAbs (IgG1) were added to the IL-15 DC cultures 1 hour prior to the addition of PBMCs. Percentages refer to the proportion of γδ T cells that proliferated within 5 days (*n* = 6). Friedman test with Dunn’s Multiple Comparison Test. **(C)** After overnight culture of γδ T cells in full PBMC fraction with IL-15 DCs + IPP (= cond) and K562 or Daudi tumor cells (E:T ratio = 1:10), with anti-IL-15 mAbs (aIL-15) or with isotype control mAbs (IgG1), intracellular IFN-γ production was measured with flow cytometry in γδ T cells (*n* = 6). Friedman test with Dunn’s Multiple Comparison Test. ** *p* < 0.01 and * *p* < 0.5; ns, *p* > 0.5.

As apparent from the previous experiments, IL-15 DCs clearly induce γδ T cell proliferation and IFN-γ production by contact-independent mechanisms. Therefore, we endeavored to identify the soluble factor(s) produced by the IL-15 DCs triggering γδ T cell activation. We previously performed a cDNA microarray analysis of (im)mature IL-15 DCs and “gold-standard” IL-4 DCs of three independent healthy controls (20). Interestingly, introducing IL-15 during *in vitro* differentiation of monocytes results in the generation of immature DCs producing this pro-inflammatory cytokine themselves. On the RNA level (GenBank ID: NM_000585), we detected a fold-change difference of 3.6 in expression signal between immature IL-15 DCs (Probe signal: 142) versus IL-4 DCs (Probe signal: 40). In concordance with these data, we have shown that mature IL-4 DCs do not secrete IL-15 (22). Subsequently, we examined the IL-15 secretion of IL-15 DCs. The concentration of IL–15 in 48-hour wash-out supernatant of 1 × 10^6^ IL-15 DCs was found to be 275 ± 107 pg/mL (Figure 3A). To clarify the involvement of this pleiotropic cytokine, IL-15 effects were canceled out using neutralizing mAbs (Figures 3B,C). IL-15 DC-mediated γδ T cell proliferation was reduced by approximately 60% upon IL-15 neutralization. Concerning IFN–γ production, blocking IL-15 significantly reduced the ability of γδ T cells to produce IFN–γ upon stimulation with IL-15 DCs in a malign environment.

### IL-15 DCs Potentiate γδ T Cells From Healthy Donors to Kill Leukemic Cell Lines and Allogeneic Primary AML Blasts

**Figure 4:**
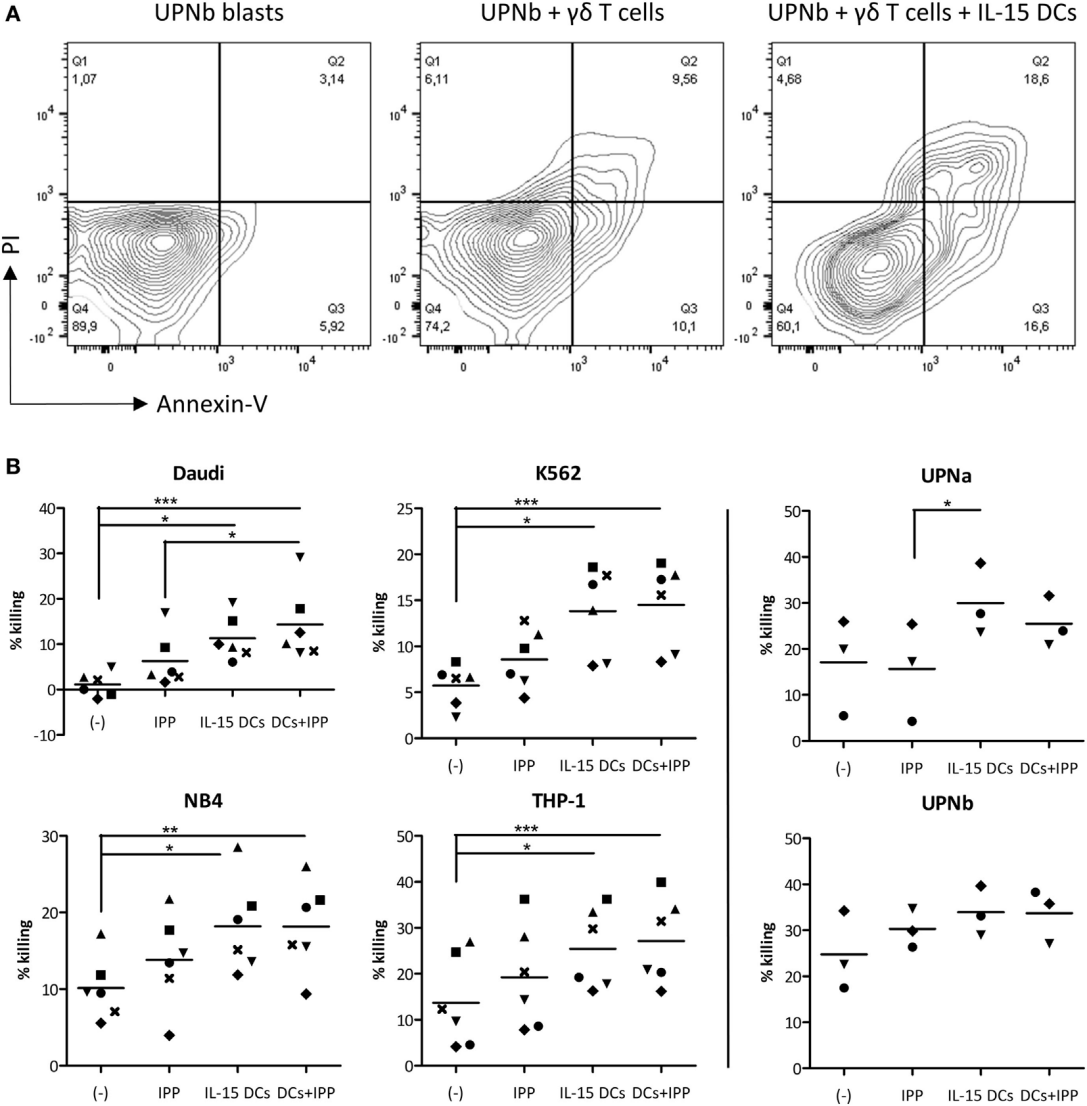
The cytotoxic capacity of γδ T cells is radically increased by IL-15 dendritic cells (DCs). **(A)** Representative example of primary acute myeloid leukemia blast killing (UPNb) from one out of three donors. **(B)** Cytotoxicity was determined of γδ cell ± IL-15 DC (co-)cultures with or without isopentenyl pyrophosphate after 24–36 hours. Unstimulated γδ T cells (−) were used to define basal killing capacity. Target cells were added at an E:T ratio of 2:1. Percentage tumor cell killing was ascertained by Annexin-V/PI staining after 4 hours and calculated using the formula specified in Section “Materials and Methods.” Donors are represented by unique symbols. Friedman test with Dunn’s Multiple Comparison Test. *** *p* < 0.001, ** *p* < 0.01, and * *p* < 0.5.

After establishing the stimulatory effect of IL-15 DCs on γδ T cell proliferation and pro-inflammatory cytokine production, we looked at their ability to improve γδ T cell lysis of a panel of leukemic tumor cell lines and primary AML blasts. As shown in Figure 4, resting (unstimulated) γδ T cells failed to lyse Daudi tumor cells (1.10 ± 1.07%) had a low cytotoxic activity against K562 (5.75 ± 2.23%) and killed the AML tumor cell lines NB4 (10.13 ± 1.67%) and THP-1 (13.71 ± 4.04%), and primary blasts (UPNa; 17.12 ± 6.07%, UPNb; 24.77 ± 4.96%) to some degree. This “limited” cytotoxic capacity is significantly enhanced against all targets by the addition of IL-15 DCs: Daudi = 11.31 ± 2.00%, K562 = 13.83 ± 1.95%, NB4 = 18.18 ± 2.49, THP-1 = 25.46 ± 3.56%, UPNa = 29.99 ± 4.48%, and UPNb = 33.93 ± 3.11%. Additional stimulation with IPP has little influence on the overall lytic activity of γδ T cells. As regards primary AML blasts of donor UPNa, even a small but noticeable negative effect on killing was observed with IPP stimulation.

### γδ T Cells From AML Patients in First Complete Remission are Robustly Activated by IL-15 DCs

Next, we sought to investigate whether γδ T cells present in the peripheral blood of treated AML patients (Table 1) could be efficiently stimulated by IL-15 DCs. Therefore, the abovementioned experiments were repeated with monocyte-depleted PBLs (containing γδ T cells) from AML patients in complete remission. Since previous experiments in healthy donors were carried out with full PBMCs, we first established the equivalence of both experimental designs with immune cells of healthy donors (Figure S4 in Supplementary Material).

At the outset, we looked at the cytotoxic phenotype of γδ T cells of AML patients (after activation) (Table 3; % and ΔMFI, Figure S3 in Supplementary Material; histogram overlays). The expression level of CD16, both basally as well as after exposure to IL-15 DCs, was similar to that of healthy donors. Interestingly, whereas IL-15 DCs of healthy volunteers did not affect the expression of NKG2D, we noticed a trend towards upregulation of this marker on γδ T cells of AML patients upon co-culture with IL-15 DCs. Of note, UPN2 had a low basal expression of both markers, but showed a similar reaction to activation as compared to γδ T cells from healthy donors. In UPN4, baseline CD16 and NKG2D expression was already high, making it difficult to observe any additional activation. Strikingly, in all patients a robust, more than 2-fold, induction of NKp30 expression on the γδ T cell surface was observed upon IL-15 DC stimulation (21.73% in IL-15-DC-stimulated γδ T cells versus 9.5% in unstimulated γδ T cells).

**Table 3:** γδ T cell phenotype of acute myeloid leukemia patients 48-hours after co-culture with IPP, IL-15 dendritic cells (DCs) or both.

%	Resting	IPP	IL-15 DCs	IPP + IL-15 DCs
CD16	25.92 ± 18.12%	25.21 ± 15.87%	31.50 ± 16.36%	33.55 ± 14.07%
NKG2D	45.91 ± 21.39%	47.51 ± 19.27%	49.27 ± 20.06%	52.79 ± 19.79%
NKp30	9.500 ± 2.359%	20.02 ± 6.94%	21.73 ± 1.24%	32.70 ± 5.20%
BTLA	59.78 ± 5.41%	66.93 ± 7.90%	60.73 ± 6.80%	65.18 ± 7.77%
PD-1	1.058 ± 0.248%	6.213 ± 4.212%	2.188 ± 0.818%	13.76 ± 6.098%*
CD80	1.465 ± 0.581%	6.670 ± 2.981%	9.875 ± 5.415%	11.45 ± 6.73%*
CD86	8.205 ± 5.252%	19.73 ± 8.87%	23.18 ± 10.54%	32.15 ± 1218%**

**ΔMFI**	**Resting**	**IPP**	**IL-15 DCs**	**IPP + IL-15 DCs**

CD16	174.7 ± 138.1	227.0 ± 176.2	280.7 ± 189.8	334.0 ± 226.1*
NKG2D	7170 ± 2522	10237 ± 3279	9177 ± 3453	12324 ± 4861
NKp30	60.75 ± 3.199	113.0 ± 35.39	132.8 ± 27.19	187.3 ± 40.16
BTLA	150.3 ± 44.83	200.4 ± 62.20	168.2 ± 55.47	197.3 ± 71.39
PD-1	16.50 ± 11.68	101.0 ± 39.79	38.75 ± 19.04	430.0 ± 290.7
CD80	10.73 ± 2.414	95.55 ± 39.21	99.48 ± 48.84	120.9 ± 59.81
CD86	29.75 ± 16.87	66.15 ± 27.68	96.40 ± 45.45	129.7 ± 46.12*

ΔMFI, difference in mean fluorescence intensity between the marker and corresponding isotype control. Asterisks indicate a statistically significant difference in cell surface marker expression between stimulated γδ T cells and resting γδ T cells (*n* = 4; Friedman test with Dunn’s Multiple Comparison Test; * *p* < 0.05, ** *p* < 0.01).

Secondly, expression of co-stimulatory and co-inhibitory molecules was examined (Table 3; % and ΔMFI). Upregulation of CD80 on the γδ T cell surface was observed in 3 of the 4 AML patients (mean; >6-fold increase in CD80 expression upon stimulation with IL-15 DCs as compared to baseline) and CD86 in all 4 patients (mean; 23.18% versus 8.21% baseline). Concerning PD-1 and BTLA, PD-1 expression was virtually absent on γδ T cells of AML patients in remission (mean; 1.058%), in contrast to healthy donors (mean; 6.738%), whereas the BTLA expression was slightly more pronounced (mean; 59.78% versus 47.78%). The expression of both molecules was not affected by IL-15 DCs, i.e. mean expression of 2.188% PD-1 and 60.73% BTLA.

For all markers the influence of the addition of IPP on their surface expression was comparable with the effect of IPP stimulation on healthy donor γδ T cells.

### Functionality of IL-15 DC-Activated γδ T Cells of AML Patients in Remission

**Figure 5:**
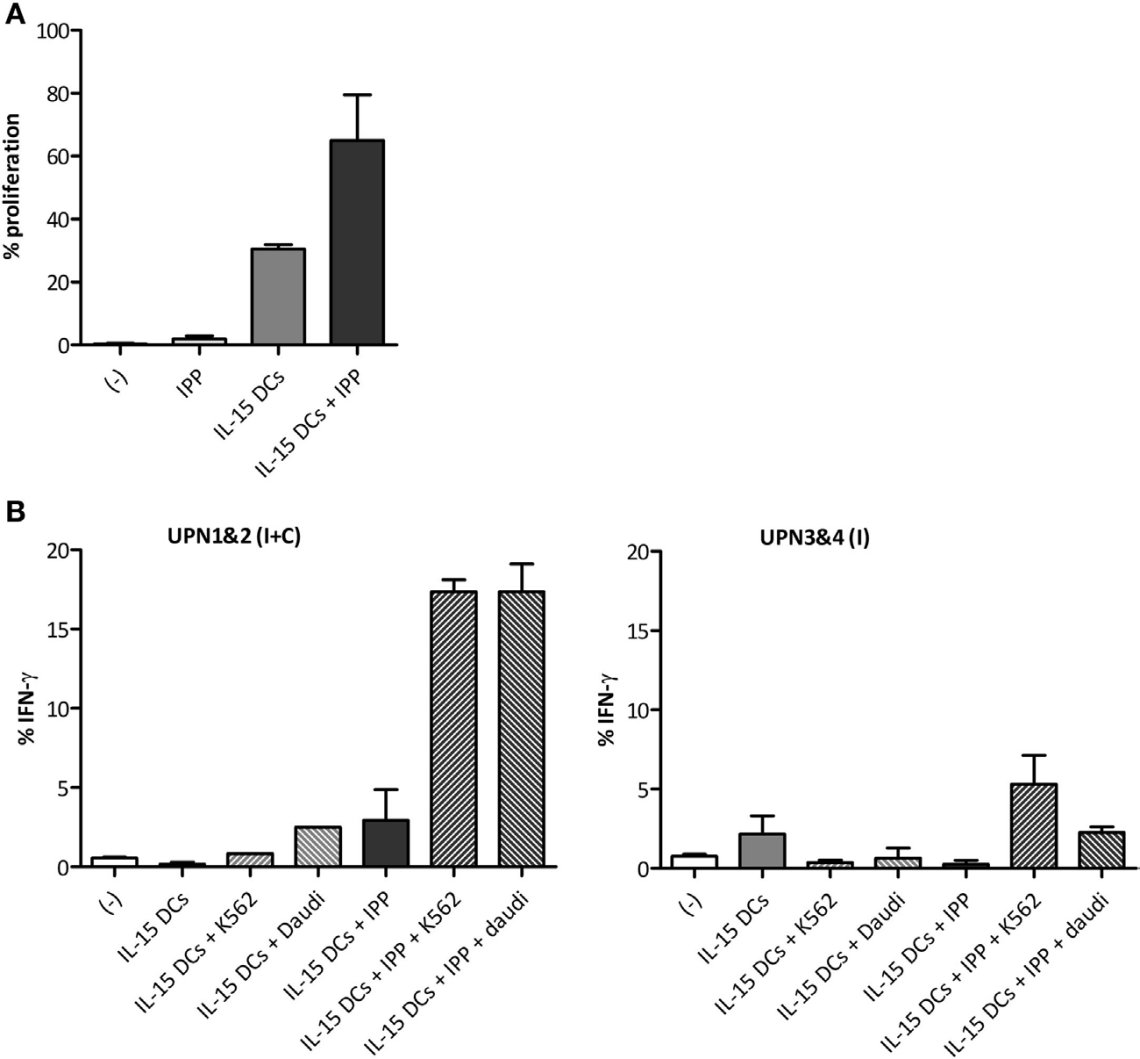
Functional activation of γδ T cells of acute myeloid leukemia (AML) patients by IL-15 dendritic cells (DCs). **(A)** Bar graphs depict γδ T cell proliferation upon 5-day culture (−) with isopentenyl pyrophosphate (IPP), IL-15 DCs (E:T ratio = 1:10) or IL-15 DCs + IPP. **(B)** Percentage IFN-γ-positive γδ T cells after overnight culture (−) with IPP, IL-15 DCs (E:T ratio = 1:10), and/or tumor cells (E:T ratio = 1:10) measured by flow cytometry for four AML patients in first complete remission. The IFN-γ production by γδ T cells of unique patient number (UPN)1&2 and UPN3&4 is shown separately, as they differ from each other in response to stimuli. Blood from UPN1&2 and UPN3&4 was drawn after termination of induction and consolidation chemotherapy (I + C) and induction chemotherapy (I), respectively. No statistics were performed due to limited numbers.

Finally, we aimed to examine the functionality of γδ T cells of AML remission patients upon IL-15 DC stimulation. The percentage of proliferated γδ T cells after 5-day co-culture with IL-15 DCs was 30.57 ± 1.33% in AML patients (Figure 5A), comparing favorably to the proliferative response in healthy donors. With regard to IFN-γ production by γδ T cells (Figure 5B), we observed an enhanced IFN-γ production, i.e. 15–20% intracellular IFN-γ expression, upon stimulation with IL–15 DCs in a leukemic environment in AML patients that were brought into complete remission after induction/consolidation chemotherapy (UPN1 and UPN2). However, in the other two patients (UPN3 and UPN4), who had only completed induction chemotherapy at the time of the blood draw, IL-15-DC stimulation on its own already led to a noticeable IFN-γ production by γδ T cells but with a diminished response to stimulation with additional IPP and tumor cells.

## Discussion

The clinical implementation of γδ T cells in cell-based cancer immunotherapy holds encouraging perspectives regarding the treatment of (advanced) malignancies (12, 25). This prospect is not a remote possibility anymore, evinced by the burst of several new biotech companies aiming at bringing γδ T cells into the clinic, including Gadeta (Utrecht, The Netherlands), GammaDelta Therapeutics (London, UK), Incysus (Hamilton, Bermuda), and Lymphact (Coimbra, Portugal). In this study, we aimed at harnessing γδ T cells in the anti-tumor immune response of DC vaccination, a promising and active immunotherapeutic modality, amongst others, used in the MRD setting of AML (6, 7). We recently showed that WT1 mRNA-electroporated conventional IL-4 DCs prevent or delay relapse in 43% of AML patients in remission after chemotherapy (7). However, since the IL-4 DC vaccine only weakly recruits γδ T cells (20) and generally fails to potentiate γδ T cell functions (8), this missing interaction could aid to optimize DC vaccine immunopotency, promoting cytotoxic and long-lasting anti-tumor immunity. Here, we show that our immunogenic IL-15 DC vaccine (18, 20) is able to activate γδ T cells (of AML patients in remission) and that this is attributable to a large extent to the secretion of IL-15.

First, we looked at the phenotypic features of γδ T cells upon interaction with IL-15-DCs. This has highlighted that γδ T cells of AML patients are prone to IL-15 DC stimulation, particularly in the presence of IPP. The latter is evidenced by the marked upregulation of NKp30 on the γδ T cell surface. This natural cytotoxicity receptor has been shown to be the main contributor to TCR-independent leukemia cell recognition by γδ T cells, even enabling resistant primary lymphocytic leukemia targeting (26). Interestingly, CD80 and CD86, specific antigen-presenting (APC) cell markers involved in co-stimulatory signaling to αβ T cells, are clearly upregulated on γδ T cells after interaction with IL–15 DCs. This could be of importance, considering γδ T cells have been shown to adopt an APC phenotype upon activation and to effectively (cross-)present peptide antigens (27, 28). Moreover, the upregulation of CD16 on the cell membrane enables antibody-dependent cellular cytotoxicity and the efficient “licensing” of γδ T-APCs (29).

Subsequently, we looked at negative co-stimulatory markers associated with T cell exhaustion, i.e. BTLA and PD-1 (30). These inhibitory molecules were not, or only to a limited extent, induced by IL-15 DCs on γδ T cells. On the other hand, we are the first to show that IPP causes PD-1 expression. This is of importance in view of the increased IPP expression by cancerous cells and the compelling evidence on the stimulatory effect of IPP for γδ T cell activation and expansion (8). Interestingly, γδ T cells are able to surmount (in part) the inhibitory effects mediated by PD-1 by means of TCR triggering (31). Indeed, sensitization of tumor cells with zoledronate, a nitrogen-containing bisphosphonate resulting in the accumulation of IPP through the inhibition of farnesyl pyrophosphate synthase (14), overcomes the inhibitory effect of PD-1. Therefore, the relationship between IPP signaling and PD-1 expression/functioning warrants further investigation. Besides, DC therapy in concert with anti-PD-1 therapeutics is a combination to be investigated, possibly rendering AML cells more susceptible to immune attack. This especially considering the expression of PD-1 ligands is a well-recognized mechanism by which AML cells inhibit anti-leukemic immune cell responses (32, 33). The results of a clinical trial combining anti-PD-1 therapeutics with a DC-based AML vaccine are therefore awaited (34).

To date, the capacity of circulating DCs to trigger γδ T cell activation is largely unknown. Notwithstanding, we and others have shown that autologous *ex vivo* generated IL-4 DCs, used routinely for clinical studies, are inefficient in mobilizing γδ T cells (20) and unable to induce γδ T cell proliferation and effector functions, and that additional/alternative signals are required (35). In this study we provide evidence that IL-15 DCs are able to induce autologous γδ T cell proliferation and a Th1-like polarization profile and that these features were conserved in AML patients who are in complete remission. Perhaps even more important, IL-15 DCs are able to significantly upgrade γδ T cell cytotoxicity against leukemic cell lines and primary AML blasts. This makes the IL-15 DC vaccine an all-round activator of the cytotoxic immune effector response, to wit γδ T cells, NK cells (19) and conventional T cells (17). The interesting observation that γδ T cells from AML patients before consolidation chemotherapy exhibited a different functional profile with regard to IFN-γ production as compared to that of patients after a consolidation regimen needs to be confirmed in a larger cohort of AML remission patients. This might highlight the importance of timing of administration of γδ T cell-activating immunotherapeutic strategies in AML (36).

Future work will also need to reveal if patients would benefit of the addition of IPP to the vaccine or if there is sufficient IPP present on the leukemic residual cells to enhance γδ T cell activation. Seminal work of Gundermann et al. has already shown that if AML cells are pretreated with zoledronate, they display a significantly augmented predisposition to γδ T cell-mediated cytotoxicity. The latter suggests a potential benefit of the addition of IPP to the vaccine or the combination with a nitrogen-containing bisphosphonate (14, 37). This is supported by our current data showing an enhanced proliferative response and IFN-γ production by γδ T cells to IL-15 DC stimulation in combination with IPP. At the same time, no apparent effect could be detected of the addition of a phosphoantigen as regards γδ T cell cytotoxicity.

Of note, phosphoantigens, such as IPP, activate γδ T cells in a TCR-mediated manner, whereby the expression of ubiquitous butyrophilin (BTN) proteins CD277/BTN3A on stimulator cells were shown to be indispensable (38–41). In accordance with other data showing that BTN3 molecules are widely expressed by immune cells, some tumor cell lines and even monocyte-derived DCs (42), it can be assumed that the presentation of IPP to γδ T cells was not hampered in our experiments. Of note, since BTN3A is a determining factor of γδ T cell-recognition of AML cells and agonistic BTN3A mAbs have been show to circumvent primary AML blast resistance to allogeneic γδ T cell lysis, combination therapy is here too something to consider (43).

Finally, focusing on the IL-15 DC-mediated γδ T cell activation itself, the stimulatory effect seemed to be mainly attributable to the secretion of IL-15 by the IL-15 DC vaccine. IL-15 is a well-documented regulator of homeostasis and activator of both innate and adaptive immunity (44). Its non-redundant role is reflected by the fact that CD8^+^ memory T cells and NK cells are absent in IL-15-deficient environments (45, 46), and the lack of IFN-γ-producing γδ T cells in IL-15^−/−^ mice (47). Moreover, the anti-tumor effect of IL-15 on the immune system has been well-documented in experimental systems (48–50). Concerning IL-15-mediated γδ T cell activation, our findings are in line with data describing γδ T cell proliferation in rhesus macaques after continuous administration of IL-15 (51) and an increase in absolute γδ T cell counts, accompanied by an upsurge in proliferating γδ T cells, in the first-in-human trial of recombinant IL-15 in cancer patients (52). Previously, we have shown that IL-15 supports γδ T cell proliferation *ex vivo* and that the addition of IL-15 to γδ T cell cultures results in a more pronounced Th1 polarization and an increased cytotoxic capacity of γδ T cells (53). In addition, pediatric thymic tissue has been used to unravel the process that drives human γδ T cell differentiation toward anti-tumor lymphocytes. They have confirmed that IL-15 signaling is sufficient to guide human γδ T cells along the Th1 pathway, coinciding with a strong killing capacity of leukemia cells (54). Moreover, the lack of IL-15 production by *M.tuberculosis*-infected DCs leads to deficient γδ T cell effector functions, impeding the conversion of central memory γδ T cells into effector memory cells (55). Although IL-15 is a promising immunotherapeutic candidate, and currently tested in clinical trials in AML (National Cancer Institute Trial IDs; 2016LS056 and 2016LS058), the stability and side effects of recombinant IL-15 are some of the bottlenecks to be overcome (44). The targeted release of IL-15 by IL-15 DCs might therefore offer an important advantage over the systemic delivery of recombinant IL-15, namely avoiding substantial toxicity (52).

In summary, we have shown that the IL-15 DCs are able to harness γδ T cells in their anti-tumoral activity *via* secretion of soluble IL-15. These data support the implementation of IL-15-expressing DCs into future clinical trials.

## Ethics Statement

This study was carried out in accordance with the recommendations of the Ethics Committee of the Antwerp University Hospital (https://www.uza.be/ethics-committee-uza) with written informed consent from all subjects. All subjects gave written informed consent in accordance with the Declaration of Helsinki. The protocol was approved by the Ethics Committee of the Antwerp University Hospital.

## Author Contributions

HVA, ZB, ES, and VVT conceived and designed the experiments. HVA and HDR performed the experiments. HVA analyzed the data. HVA, SA, ES, and VVT wrote the paper. All authors have read and approved the manuscript in its current form.

## Conflict of Interest Statement

The authors declare that the research was conducted in the absence of any commercial or financial relationships that could be construed as a potential conflict of interest.
