# Supplementary material for: Interleukin-15-Cultured Dendritic Cells Enhance Anti-Tumor Gamma Delta T Cell Functions through IL-15 Secretion
Source: Front Immunol. 2018 Apr 10;9:658. doi: 10.3389/fimmu.2018.00658 (PMC5902500; doi:10.3389/fimmu.2018.00658)
Supplement: Supplementary file 1 [file Data_Sheet_1.PDF]

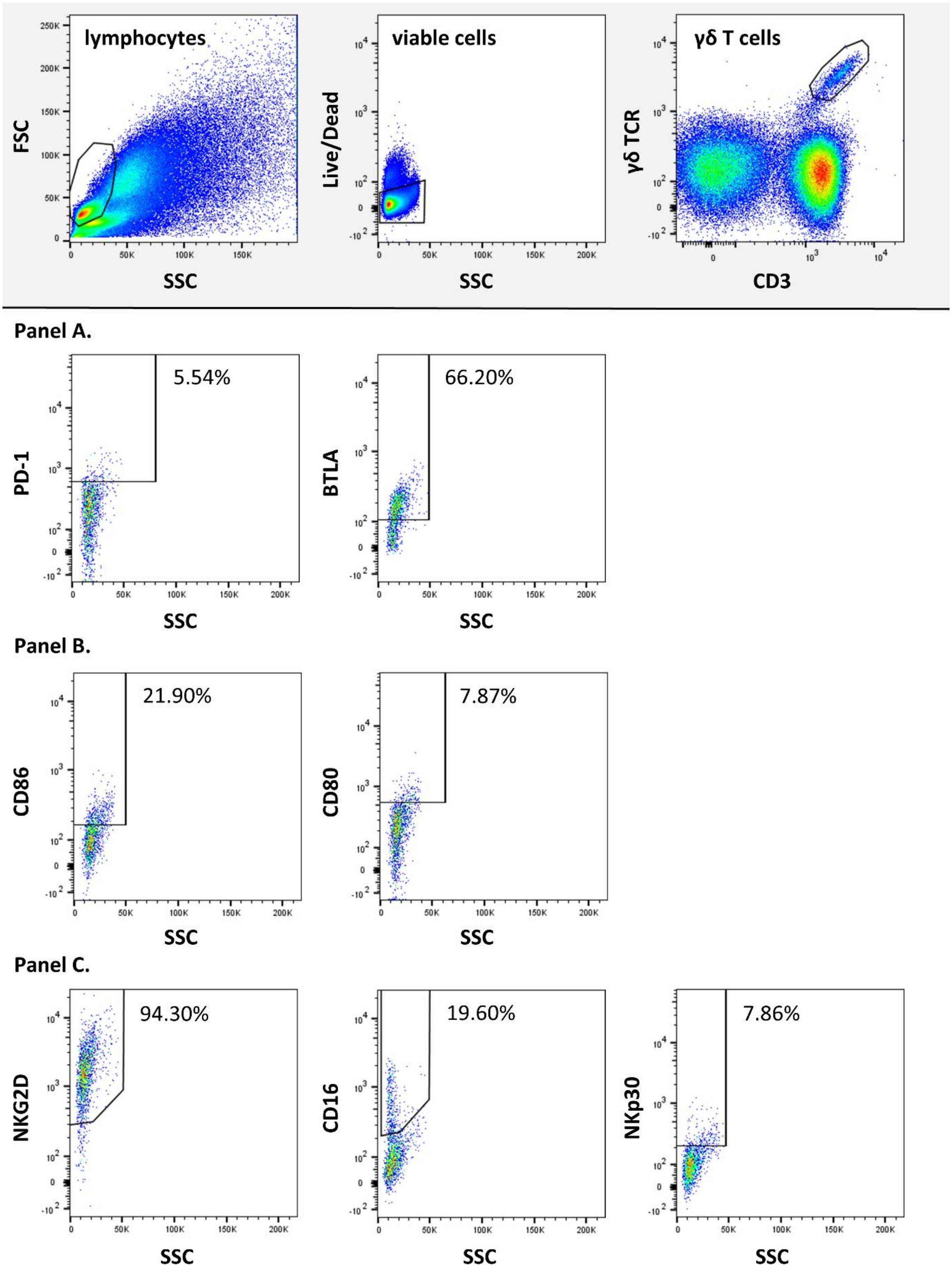

Supplementary figure 1. Gating strategy determing the phenotype of γδ T cells within unfractionned PBMC. Initial gating is done on FSC and SSC, selecting lymphocytes and eliminating debris, followed by the exclusion of dead cells. Subsequently, γδ T cells are identified based on their expression of CD3(-APC-H7) and γδ TCR(-FITC [Panel C] and – APC [Panel A-B]). Phenotypic markers were assessed on the membrane of γδ T cells; Panel A) BTLA and PD-1, Panel B) CD80 and CD86 and Panel C) CD16, NKG2D and NKp30. The dot plots represent the phenotype of IL-15 DC-stimulated γδ T cells.

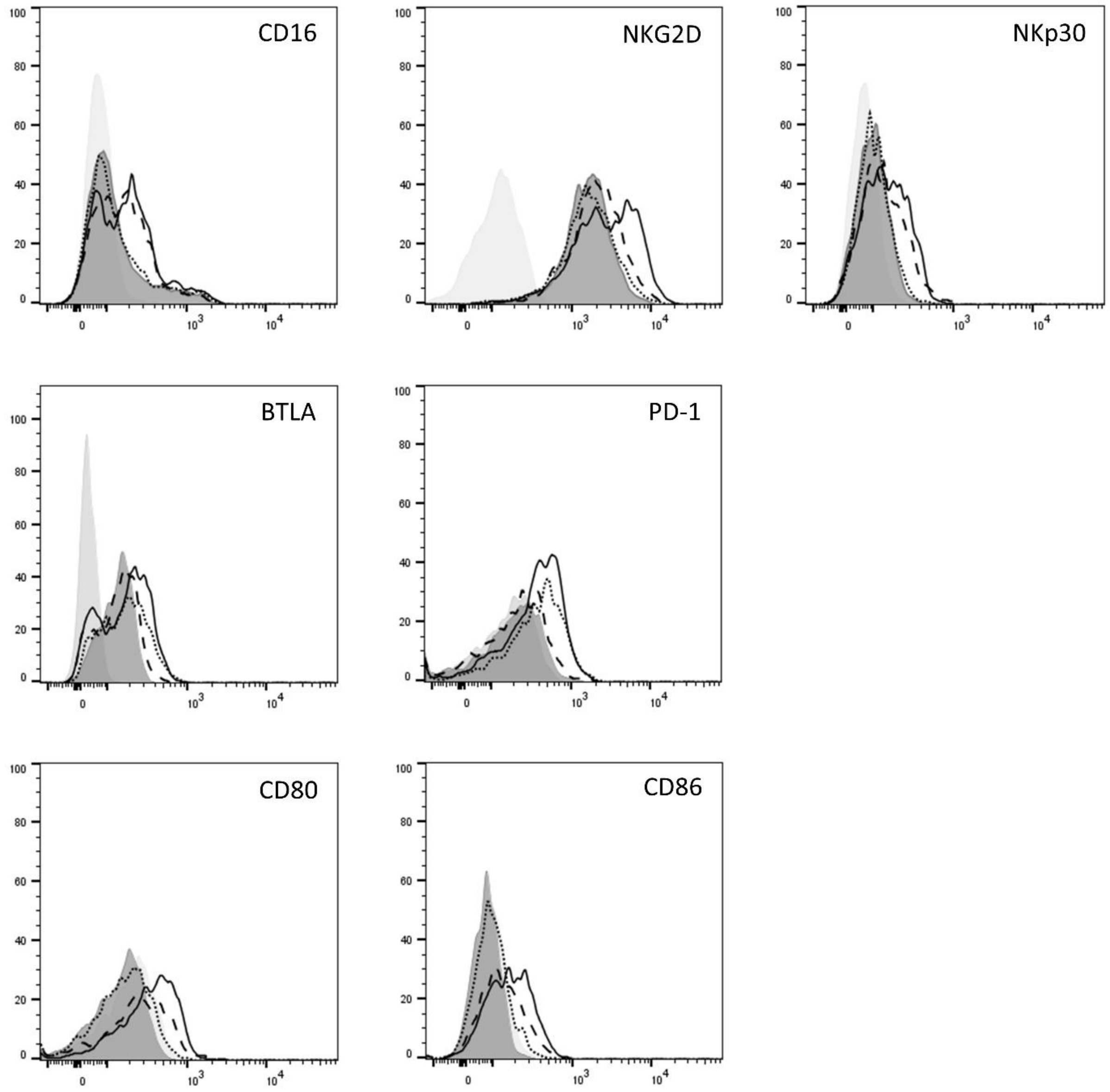

Supplementary figure 2. Flow cytometric comparison of the cell surface phenotype of γδ T cells after 48-hour culture of one healthy representative donor. Histogram overlays consist of γδ T cells within unstimulated PBMCs (filled dark grey, [filled light grey = isotype]), PBMCs stimulated with IPP (dotted line), IL-15 DCs (dashed line) and IL-15 DCs + IPP (full line).

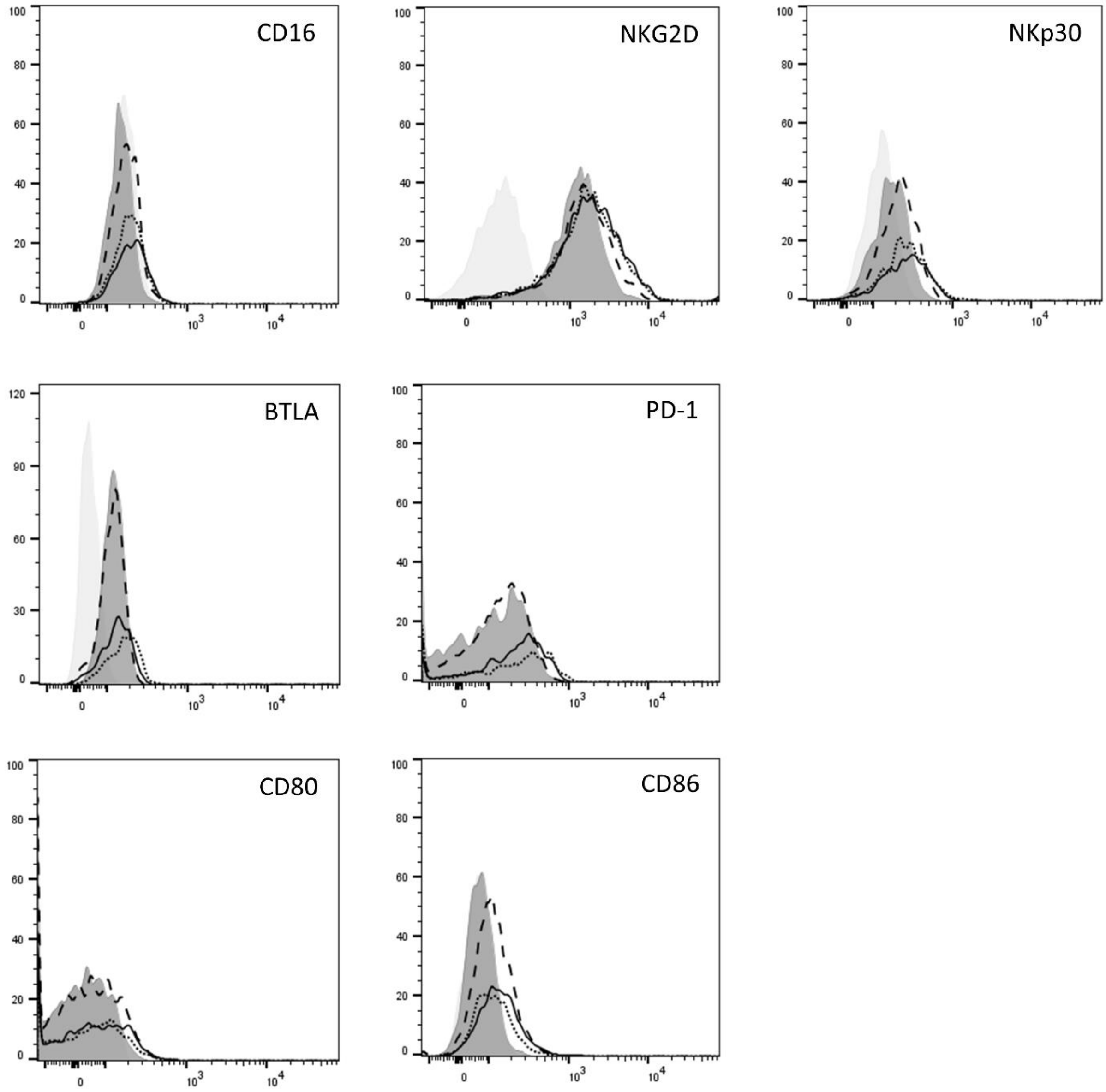

Supplementary figure 3. Flow cytometric comparison of the cell surface phenotype of γδ T cells after 48-hour culture of UPN2. Histogram overlays consist of γδ T cells within unstimulated PBMCs (filled dark grey, [filled light grey = isotype]), PBMCs stimulated with IPP (dotted line), IL-15 DCs (dashed line) and IL-15 DCs + IPP (full line).

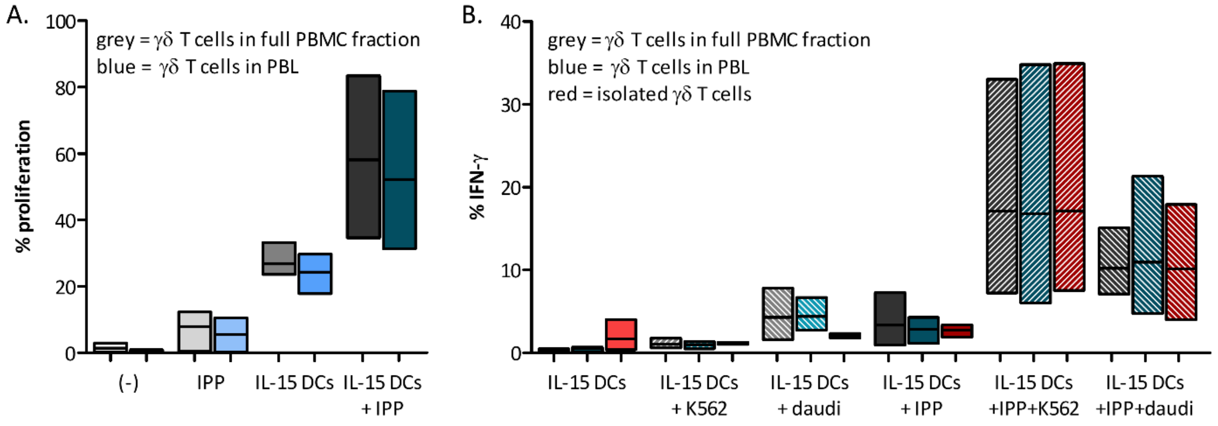

Supplementary figure 4. Direct comparison between γδ T cell activation in full fraction PBMC, CD14^{+} cell-depleted PBMC (PBL) and isolated γδ T cells. (**A**) Proliferative response of γδ T cells after 5-day culture (n = 5) and (**B**) intracellular IFN-γ production after overnight culture (n = 3), presented as floating bars (min to max - line at mean).
